# Local Entanglement of Electrons in 1D Hydrogen Molecule

**DOI:** 10.3390/e25091308

**Published:** 2023-09-08

**Authors:** Ivan P. Christov

**Affiliations:** Physics Department, Sofia University, 1164 Sofia, Bulgaria; ivan.christov@phys.uni-sofia.bg

**Keywords:** quantum entanglement, hydrogen molecule, quantum Monte Carlo

## Abstract

The quantum entanglement entropy of the electrons in a one-dimensional hydrogen molecule is quantified locally using an appropriate partitioning of the two-dimensional configuration space. Both the global and the local entanglement entropy exhibit a monotonic increase when increasing the inter-nuclear distance, while the local entropy remains peaked in the middle between the nuclei with its width decreasing. Our findings show that at the inter-nuclear distance where a stable hydrogen molecule is formed, the quantum entropy shows no peculiarity thus indicating that the entropy and the energy measures display different sensitivity with respect to the interaction between the two identical electrons involved. One possible explanation is that the calculation of the quantum entropy does not account explicitly for the distance between the nuclei, which contrasts to the total energy calculation where the energy minimum depends decisively on that distance. The numerically exact and the time-dependent quantum Monte Carlo calculations show close results.

## 1. Introduction

The entanglement in complex quantum systems manifests itself as non-separability of the many-body quantum state that occurs due to either classical interaction (e.g., Coulomb repulsion between the particles) or as a result of intrinsically quantum non-local effects (e.g., the symmetry of the wave function) [1]. Since in the former the strength of the Coulomb interaction between the particles varies in space, it makes sense to ask the question of what the spatial dependence of the entanglement is as a function of the localization of the particles. Basically, the entanglement is quantified using “global” quantum entropy, which ultimately boils down to a number where the larger the number the larger the entanglement. It would be expected however that the spatial variations of the interaction strength between the electrons would result in spatially varying entanglement. The intrinsically quantum nonlocality, on the other hand, dictates that the localization of the quantum entropy is to be considered in a configuration space where the many-body wave function resides, rather than in physical space. Previous approaches to localize entropy include the application of Bader’s concept of “atoms in molecule” where the local information entropy is calculated in terms of electron density distribution [2]. Other research investigates the localization of the entanglement of two interacting distinguishable particles in the configuration space by sampling a preliminary calculated reduced density matrix for known wave functions over a uniform grid and next calculating the von Neumann entropy [3]. It is also worth mentioning the use of the Tsallis approach to characterize the entropy properties of a single hydrogen molecule as well as of systems of three and more such molecules [4]. From the chemistry viewpoint, the local entanglement entropy considered as information entropy may find application in analysis of electron density and as correlation measure in atoms and molecules where mostly global measures have been used so far [5,6].

The primary goal of this work is to introduce a method for describing the quantum entanglement *locally* by using a localized quantum entanglement entropy as a function of the distance between two one-dimensional hydrogen atoms during their transition from separate species to the formation of a hydrogen molecule at a ground state, and beyond. Unlike in a helium atom where the s-state (opposite spin) electrons occupy essentially the same space, in the hydrogen molecule there is an axis of symmetry and the question about the entanglement fluctuations in space makes more sense, especially for identical particles. Using the direct numerical solution of the two-body time-dependent Schrödinger equation it is shown that the reduced density matrix (RDM) for each of the electrons can be found using a set of Monte Carlo walkers, which sample the probability distribution in the 2D configuration space. The RDM is next used to find the quantum entanglement entropy as an approximation to the von Neumann quantum entropy, named linear entropy, calculated at appropriate regions in the configuration space oriented along the separation between the atoms. It is found that the quantum entanglement entropy exhibits its maximum in the middle between the atoms, even when those are well separated.

Since, in general, the numerical solution of the Schrödinger equation suffers an exponential scaling of the computational resources with the number of particles, here we also apply the recent time-dependent quantum Monte Carlo (TDQMC) method, which reduces the many-body quantum problem of to a set of problems for particles and waves, which are defined in physical space-time [7,8,9,10]. This is carried out in TDQMC by introducing, for each electron, an ensemble of point-like walkers in space and a concurrent ensemble of guide waves as walkers in the “waves space”, where these two ensembles are mutually connected through the set of Schrödinger-type equations, together with guiding equations or with a combined drift-diffusion process. Through the particle-wave dichotomy, the particles react to the wave’s evolution while, at the same time, the waves experience a back reaction from the particles’ motion, in a self-consistent manner. The main advantage of using the TDQMC formalism is that the quantum correlations, which are due to interaction potentials between the particles, can be accounted for in a tractable way, even for many-body systems. The TDQMC method scales almost linearly with the number of particles for bosonic states and scales, in general, no worse than time dependent Hartree-Fock.

The results of the present work would allow one to consider in more detail the quantum information processes of formation of molecules and it can easily be extended to more complex structures such as clusters, nanostructures, etc., in higher spatial dimensions.

## 2. Methods

First, we calculate the ground state of two one-dimensional hydrogen atoms with coordinates of their nuclei $X_{1},X_{2}$, by solving the time dependent Schrödinger equation in imaginary time t=−iτ (in atomic units)

(1) $$-\frac{\partial }{\partial \tau }\Psi (x_{1},x_{2},\tau )=H\left(x_{1},x_{2}\right)\Psi (x_{1},x_{2},\tau ),$$

where the Hamiltonian reads

(2) $$H\left(x_{1},x_{2}\right)=-\frac{1}{2}\left(\frac{\partial^{2}}{\partial x_{1}^{2}}+\frac{\partial^{2}}{\partial x_{2}^{2}}\right)+\sum_{i,j}^{2}V_{e-n}(x_{i}-X_{j})+V_{e-e}(x_{1}-x_{2}),$$

and the imaginary time propagation in Equation (1) ensures that for an arbitrary initial wave function the higher order states are projected out as steady state is established (see, e.g., [11]).

To avoid numerical complications due to the singularity of the Coulomb potential at the position of the nuclei, it is assumed that the electron-nuclear and electron–electron interactions are approximated by modified potentials [12]:

(3) $$V_{e-n}(x_{i}-X_{j})=-\frac{1}{\sqrt{1+{\left(x_{i}-X_{j}\right)}^{2}}};$$

(4) $$V_{e-e}(x_{i}-x_{j})=\frac{1}{\sqrt{1+{\left|x_{i}-x_{j}\right|}^{2}}},$$

where *i*, *j* = 1, 2, and where in Equations (1) and (2), we treat the nuclei as classical particles. This is justified within our approach of gradually changing the distance between the nuclei and next calculating the ground state of the electronic system, without accounting for the nuclear dynamics explicitly. The ground-state wave function was found either by solving directly Equation (1) using, e.g., the split-step Fourier method or by applying the time-dependent quantum Monte Carlo method, which introduces concurrent ensembles of walkers and guide waves defined in physical space where the many-body problem is reduced to a set of coupled Schrödinger-type equations for the guide waves $\varphi_{i}^{k}(x_{i},\tau )$ for the *i*-th electron; *k =* 1, 2,…, *M* denotes the different walkers [7,8,9,10]:

(5) $$-\frac{\partial }{\partial \tau }\varphi_{i}^{k}(x_{i},\tau )=\left[-\frac{1}{2}\frac{\partial^{2}}{\partial x_{i}^{2}}+V_{e-n}\left(x_{i},X_{j}\right)+V_{eff}^{k}\left(x_{i},\tau \right)\right]\varphi_{i}^{k}(x_{i},\tau );\;i\;=\;1,2$$

where $V_{eff}^{k}\left(x_{i},\tau \right)$ is the effective electron-electron interaction potential given by a Monte Carlo convolution of the true interaction potential $V_{e-e}\left(x_{i}-x_{j}\right)$ of Equation (4) and a kernel function $Κ\left[x_{j},x_{j}^{k}(\tau ),\sigma_{j}\right]$, which accounts for the spatial nonlocality experienced by each walker due to the quantum uncertainty:

(6) $$V_{eff}^{k}\left(x_{i},\tau \right)=\sum_{j\neq i}^{2}\frac{1}{Z_{j}^{k}}\sum_{l}^{M}V_{ee}\left[x_{i},x_{j}^{l}(\tau )\right]Κ\left[x_{j}^{l}(\tau ),x_{j}^{k}(\tau ),\sigma_{j}\right];$$

(7) $$Κ\left[x_{j},x_{j}^{k}(\tau ),\sigma_{j}\right]=\exp\left(-\frac{{\left|x_{j}-x_{j}^{k}(\tau )\right|}^{2}}{2\sigma_{j}{}^{2}}\right),$$

where *i*, *j* = 1,2, and $\sigma_{j}$ is the characteristic length of spatial nonlocality, which is numerically close to the standard deviation of the *j*-th walkers distribution [13] and which variationally minimizes the energy between the limiting cases of pairwise interaction between the walkers ($\sigma_{j}\to 0$, described by a set of linear equations [8]), and the Hartree-Fock approximation ($\sigma_{j}\to \infty$), which is essentially nonlinear with respect to the wave functions. Notice that the waves $\varphi_{i}^{k}(x_{i},\tau )$ considered as random variables in TDQMC do not have their own ontological meaning and similarly the ground-state energies of Equation (5) are not directly related to the ground-state energy of the electron. Notice also that although employing particles and waves, the TDQMC method differs from Bohmian mechanics, which is an exact theory relying on the many-body wavefunction, and as such, it experiences the exponential scaling with the number of physical particles, while the TDQMC method scales almost linearly for opposite-spin electrons.

For imaginary-time propagation the trajectories $x_{i}^{k}(\tau )$ are determined by a drift-diffusion process [9]:

(8) $$dx_{i}^{k}\left(\tau \right)=v^{D}{}_{i}^{k}d\tau +\eta_{i}\left(\tau \right)\sqrt{d\tau },$$

where

(9) $$v^{D}{}_{i}^{k}\left(\tau \right)={\left.\frac{\nabla_{i}\varphi_{i}^{k}(x_{i},\tau )}{\varphi_{i}^{k}(x_{i},\tau )}\right|}_{x_{i}=x_{i}^{k}(\tau )}$$

is the drift velocity whenever birth–death of walkers is applied, and $\eta_{i}\left(\tau \right)$ is a Markovian stochastic process. At the same time, the walkers $x_{i}^{k}(\tau )$ sample the moduli square of the corresponding guide waves ${\left|\varphi_{i}^{k}(x_{i},\tau )\right|}^{2}$.

Here, we apply Equations (1)–(9) to find both the global and the local quantum entanglement entropy of the electrons in 1D hydrogen molecule, where the ground state is entangled due to the Coulomb interaction between the electrons considered as identical particles. As a measure of entanglement, we first use the *global* linear quantum entropy S(τ) calculated using the reduced density matrix for each of the two electrons in the molecule:

(10) $$S\left(\tau \right)=Tr\left[\rho -\rho^{2}\right]=1-\int \rho^{2}\left(x,x,\tau \right)dx,$$

where for the exact reduced density matrix, we have (for normalized wavefunctions)

(11) $$\rho_{i}^{E}\left(x_{i},x_{i}^{\prime },\tau \right)=\int \Psi (x_{i},x_{2},\tau )\Psi^{*}(x_{i}^{\prime },x_{2},\tau )dx_{2}$$

On the other hand, the guide waves provided by the TDQMC method for the *i*-th electron (Equation (5)) can be used to efficiently calculate the one-body density matrix considered as covariance matrix for the random variables $\varphi_{i}^{k}$ [14], without the need to calculate the density matrix of the whole system:

(12) $$\rho_{i}^{TDQMC}=E\left(\varphi_{i}^{*}\varphi_{i}\right)=\int P\left[\varphi_{i}\right]\varphi_{i}^{*}\varphi_{i}D\varphi_{i}^{*}D\varphi_{i}$$

where the random state $\varphi_{i}^{k}$ is defined in terms of the probability distribution $P\left[\varphi_{i}\right]$. Assuming that, according to the particle-wave dichotomy [9], the probability distribution of the waves $\varphi_{i}^{k}$ corresponds to the distribution of the walkers $x_{i}^{k}$, we arrive at the TDQMC reduced density matrix

(13) $$\rho_{i}^{TDQMC}\left(x,x^{\prime }\right)=\frac{1}{M}\sum_{k=1}^{M}\varphi_{i}^{k}{}^{*}(x)\varphi_{i}^{k}(x^{\prime })$$

which, in fact, is nothing more than a Monte Carlo sum representation of the integral in Equation (12). It is seen from Equation (13) that the density matrix is normalized to unity trace as long as the states $\varphi_{i}^{k}$ are normalized.

## 3. Results

Henceforth, we shall assume that the ground state has been established and we will therefore omit the time variable τ. It is seen from Equation (13) that the RDM provided by the TDQMC method is calculated through the wavefunctions of the different walkers (the upper index *k*). To compare the TDQMC RDM with the exact results for a 1D hydrogen molecule, we should calculate the exact density matrix in a similar way. If we assume that the two-body probability density ${\left|\Psi (x_{1},x_{2})\right|}^{2}$ is sampled by another set of Monte Carlo walkers ($x_{1}{{}^{\prime }}^{k},x_{2}{{}^{\prime }}^{k}$), we may easily calculate the reduced wave functions:

(14) $$\psi_{1}^{k}\left(x_{1}^{\prime }\right)=N_{1}\Psi \left(x_{1}^{\prime },x_{2}^{\prime }{}^{k}\right),$$

(15) $$\psi_{2}^{k}\left(x_{2}^{\prime }\right)=N_{2}\Psi \left(x_{1}^{\prime }{}^{k},x_{2}^{\prime }\right),$$

where $N_{1,2}$ are normalization factors. The wave functions in Equations (14) and (15) can be next used to calculate the reduced density matrix (also named the conditional density matrix [15]), which can also be considered exact since it is based on the numerically exact two-body wave functions in Equations (14) and (15):

(16) $$\rho_{i}^{C}\left(x_{i},x_{i}{}^{\prime }\right)=\frac{1}{M}\sum_{k=1}^{M}\psi_{i}^{k}{}^{*}(x_{i})\psi_{i}^{k}(x_{i}{}^{\prime })$$

Although the calculation of the reduced density matrix in Equations (14)–(16) seems awkward, the use of walkers is important in calculating the local entanglement below.

**Figure 1:**
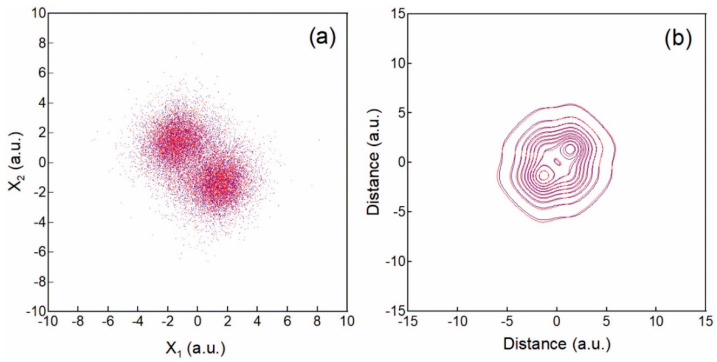
Walker distribution in configuration space for molecule of two 1D hydrogen atoms at a distance 3a.u. (**a**); contour maps of the reduced density matrix (**b**). Blue lines and points—exact result; red lines and points—from TDQMC.

We start with the calculation of the walker’s distributions and the reduced density matrices for a fixed inter-nuclear distance $\left|X_{1}-X_{2}\right|=3a.u.$ Figure 1b presents the contour-line images of the exact RDM $\rho_{}^{E}\left(x,x^{\prime }\right)$ from Equation (11) (blue line) as compared to the TDQMC RDM $\rho_{}^{TDQMC}\left(x,x^{\prime }\right)$ from Equation (13) (red line) where the two electron distributions are at their ground state (Figure 1a). It is seen that there is a good correspondence between the exact and the TDQMC results, which clearly reflect the symmetry of the ground state.

Next, we calculate the ground-state energy of the two-atom configuration as function of the distance between the nuclei [16];

(17) $$E_{1}=\frac{1}{M}{\sum_{k=1}^{M}\left[\sum_{i=1}^{2}\left[-\frac{1}{2}\frac{\nabla_{i}^{2}\varphi_{i}^{k}(x_{i}^{k})}{\varphi_{i}^{k}(x_{i}^{k})}+V_{e-n}(x_{i}^{k})\right]+\sum_{i>j}^{2}V_{e-e}(x_{i}^{k},x_{j}^{k})\right]}_{\begin{array}{l} x_{i}^{k}=x_{i}^{k}(\tau )\\ x_{j}^{k}=x_{j}^{k}(\tau ) \end{array}}+V_{n-n}\left(X_{1}-X_{2}\right)$$

for the TDQMC calculation, and

(18) $$\begin{array}{l} E_{2}=\iint \left[-\frac{1}{2}\Psi^{*}\left(x_{1},x_{2}\right)\left(\frac{\partial^{2}}{\partial x_{1}^{2}}+\frac{\partial^{2}}{\partial x_{2}^{2}}\right)\Psi \left(x_{1},x_{2}\right)\right]dx_{1}dx_{2}\\ +\iint \left[\sum_{i,j}^{2}V_{e-n}(x_{i}-X_{j})+V_{e-e}(x_{1}-x_{2})\right]{\left|\Psi \left(x_{1},x_{2}\right)\right|}^{2}dx_{1}dx_{2}\\ +V_{n-n}\left(X_{1}-X_{2}\right) \end{array}$$

for the exact two-electron problem. In Equations (17) and (18), $V_{n-n}\left(X_{1}-X_{2}\right)$ is the soft nucleus–nucleus potential:

(19) $$V_{n-n}\left(X_{1}-X_{2}\right)=\frac{1}{\sqrt{0.5+{\left(X_{1}-X_{2}\right)}^{2}}}$$

**Figure 2:**
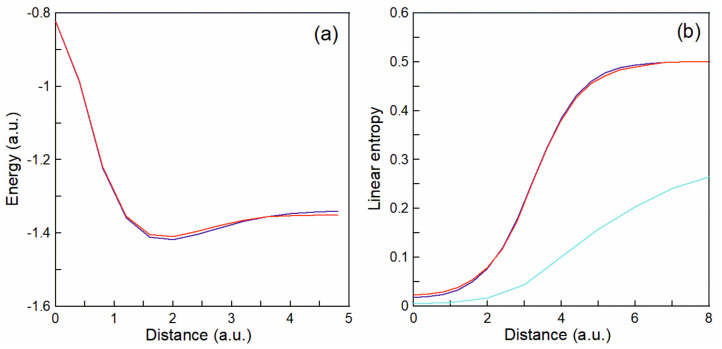
Ground state energy of hydrogen molecule (**a**) and global linear entropy (**b**) as function of the internuclear distance. Blue lines—exact result; red lines—from TDQMC. The green line in (**b**) shows the peak value of the local entropy, multiplied by 10.

Figure 2a shows the minimum of the energy that occurs at a distance $\left|X_{1}-X_{2}\right|∼2a.u.$ between the nuclei where a stable hydrogen molecule is formed for both the exact (blue line) and TDQMC (red line) calculations. Figure 2b shows with red and blue lines, the global linear entropy as predicted by Equation (10) as a function of the inter-nuclear distance, for the two electrons considered as identical particles (see Figure 1a). It is seen that the TDQMC prediction from Equations (10) and (13) (red line) is in good agreement with the numerically exact result of Equations (10) and (11) (blue line). The red and blue curves in Figure 2b are qualitatively similar to the result in [17] where the configuration interaction method has been used to estimate the global entanglement of the hydrogen molecule as function of the inter-nuclear separation.

**Figure 3:**
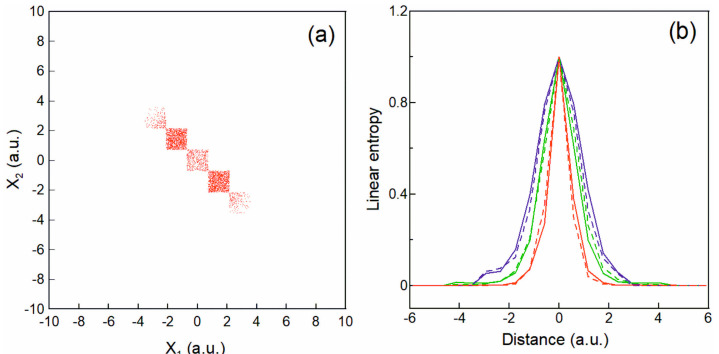
Partition of the 2D configuration space used in the calculations (**a**); (**b**) local linear entropy for different inter-nuclear distance d: d = 0—blue line; d = 3a.u.—green line; and d = 5a.u.—red line. Solid lines—exact results; dashed lines—TDQMC results.

Next, we focus on our approach to calculate the *local* entanglement at different regions in configuration space $\left(x_{1},x_{2}\right)$. Figure 3a depicts the partition we use here represented as a set of square regions along the diagonal of the walker’s distribution of Figure 1a where the global reduced density matrix of Equation (13) can be represented as a sum:

(20) $$\rho_{i}^{TDQMC}\left(x,x^{\prime }\right)=\sum_{m=1}^{N}\rho_{i}^{m}=\sum_{m=1}^{N}\frac{1}{M_{m}}\sum_{k=1}^{M_{m}}\varphi_{i}^{k}{}^{*}(x)\varphi_{i}^{k}(x^{\prime }),$$

where *N* is the total number of square regions in Figure 3a, and $M_{m}$ is the number of walkers within the *m*-th square region. Notice that although the density matrix can always be represented as the sum in Equation (20), the quantum entropy, which is a nonlinear function of the density matrix, is not additive. In order to cover the density distribution adequately, we use 50 regions in Figure 3a with a total of 200,000 walkers for each electron. Then, we calculate the local entanglement using *local* linear entropy introduced according to:

(21) $$S_{i}^{m}=\int \left[\rho_{i}^{m}\left(x,x\right)-\rho_{i}^{m}{}^{2}\left(x,x\right)\right]dx,$$

where the standard normalization for the local density matrices $Tr\left[\rho_{i}^{m}\right]=1$ is in place. The choice of the entropy measure in Equation (21) ensures invariance of the local entanglement with respect to the size of the partition of configuration space in Figure 3a, as well as to the number of walkers in the total sample (Figure 1a), which contrasts with other research where the local entropy depends strongly on the size of the regions in configuration space [3]. Clearly the choice of the partition regions shown in Figure 3a is not unique, however, it proves to be a good choice in our case where the distance between the nuclei is to be varied.

Figure 3b shows the local linear entropy (from Equation (21)), normalized to unity, for different inter-nuclear distance $d=\left|X_{1}-X_{2}\right|$, from the TDQMC calculation (dashed lines) to be compared with the exact calculation (solid lines) where the representation in Equation (20) is also applied to the conditional density matrix (Equation (16)). It is seen from Figure 3b that the maximum of the local entropy is located right in the middle between the two hydrogen atoms while its width decreases by almost a factor of two between distances between the atoms d = 0 (blue line) and d = 5a.u. (red line). Also, it was found that the peak value of the local entropy (without normalization) monotonically increases from d = 0 to d = 5a.u. as is shown by the green line in Figure 2b. These findings indicate that within our approach, the local entanglement of the two electrons is localized in the middle between the two nuclei, and it becomes narrower with increasing the nucleus-nucleus separation, while its peak value increases.

## 4. Conclusions

Here, the global and the local entanglement of the electrons in a simple hydrogen molecule is quantified as a function of the distance between their nuclei. The results from the exact numerical solution of the two body Schrödinger equation are compared with those from the time-dependent quantum Monte Carlo method, which essentially reduces the quantum many-body problem from configuration space to physical space. The reduced density matrix of each of the two identical electrons is calculated in the standard way, which shows a monotonic increase in the global linear quantum entropy as the two hydrogen atoms move apart. To address the question of what the local dependence of the quantum entropy would be, we design a special partition of part of the configuration space along the orientation direction of the two-electron configuration and employ a set of particles (walkers) to sample the two-body probability distribution at the different regions. As a result, the “global” density matrix can be represented as a sum of “local” density matrices attached to each of the different regions in configuration space, where we were able to quantify the quantum entropy and hence the entanglement locally. Our findings reveal first that both the exact and the TDQMC calculation provide close results for the reduced density matrix, while at the same time, the TDQMC method scales much more favorably as compared to the exact solution. The essential result here is that unlike in atoms, the entanglement of identical electrons in a hydrogen molecule becomes localized not at the positions of the nuclei but in the middle between them, as the two atoms move apart. That result was confirmed by the two independent methods used and it is somewhat unexpected in the light of the spreading of the wave function of each electron in the molecule, which makes it less localized in space.

## Data Availability

Not applicable.
